# Lipid Accumulation in Peripheral Blood Dendritic Cells and Anticancer Immunity in Patients with Lung Cancer

**DOI:** 10.1155/2018/5708239

**Published:** 2018-04-11

**Authors:** Ryo Arai, Sayo Soda, Tomoko Okutomi, Hiroko Morita, Fumito Ohmi, Tomoe Funakoshi, Akihiro Takemasa, Yoshiki Ishii

**Affiliations:** Department of Pulmonary Medicine and Clinical Immunology, Dokkyo Medical University School of Medicine, 880 Kitakobayashi, Mibu, Tochigi 321-0293, Japan

## Abstract

We studied the subsets of peripheral blood dendritic cells (DCs) and lipid accumulation in DCs to investigate the involvement of DCs in the decreased anticancer immunity of advanced lung cancer patients. We analyzed the population of DC subsets in peripheral blood using flow cytometry. We then determined lipid accumulation in the DCs using BODIPY 650/665, a fluorophore with an affinity for lipids. Compared with healthy controls, the number of DCs in the peripheral blood of treatment-naive cancer patients was significantly reduced. In patients with stage III + IV disease, the numbers of myeloid DCs (mDCs) and plasmacytoid DCs were also significantly reduced. Lipid accumulation in DCs evaluated based on the fluorescence intensity of BODIPY 650/665 was significantly higher in stage III + IV lung cancer patients than in the controls. In the subset analysis, the fluorescence was highest for mDCs. The intracellularly accumulated lipids were identified as triglycerides. A decreased mixed leukocyte reaction was observed in the mDCs from lung cancer patients compared with those from controls. Taken together, the results show that lung cancer patients have a notably decreased number of peripheral blood DCs and their function as antigen-presenting cells is decreased due to their high intracellular lipid accumulation. Thereby, anticancer immunity is suppressed.

## 1. Introduction

Lung cancer has a poorer prognosis compared to other cancers. The pathologic analysis of tumor immunity in patients with lung cancer is important for the advancement of immunotherapy. Although treatments based on a dendritic cell (DC) vaccine for immunogenic malignant tumors, such as melanoma and renal cell carcinoma, are considered promising [1, 2], clinical trials involving lung cancer patients have also shown good outcomes [3–6]. DCs play an important role in the immune response as they are antigen-presenting cells that infiltrate cancer tissues, consistently activate cancer-specific T helper cells and cytotoxic T lymphocytes (CTL), and mediate the early stage of the antitumor response.

Human-derived DCs are typically classified into two types, myeloid DCs (mDCs) and plasmacytoid DCs (pDCs) [7, 8]. mDCs are derived from monocytes in the peripheral blood and are differentiated by the influence of granulocyte/macrophage colony-stimulating factor and interleukin- (IL-) 4, and they preferentially induce mature T helper 1 (Th1) cells, from naive T cells [9–12]. In vivo, mDCs possess strong phagocytotic and antigen-presenting abilities and are involved in the acquisition of cell-mediated immunity. Meanwhile, pDCs are induced by culture with IL-3 and mediate immunotolerance [13, 14].

DCs exist in the peripheral blood in an immature state and capture and recognize specific tumor antigens. They respond to inflammatory mediators such as interferon-alpha and toll-like receptor (TLR) agonists, and when they mature, they present antigens to T cells and acquire the ability to activate a specific antitumor T cell response and migrate to other tissues [15, 16]. DCs produce biologically active IL-12 p70 inducing a remarkable anticancer immunity by potentiating the activity of natural killer cells and inducing a Th1 response and tumor-specific CD8(+) cells [17, 18].

It is known that anticancer immunity is reduced in patients with cancer. Several tumors produce cytokines and other factors that suppress the maturation or differentiation of DCs in order to avoid the immune response [15]. CTL, which are activated by Th1-derived cytokines, show cytotoxic activity and induce apoptosis in cancer cells, but T cell activation is reduced in cancer patients, and one possible mechanism by which this occurs is through transforming growth factor-*β* (TGF-*β*), which is released by tumor cells and suppresses the activation of Th1 cells and CTL [19, 20].

Pathological impairment of DC function is considered to be a cause of decreased tumor immunity in cancer patients. All the causes of DC dysfunction have not been sufficiently elucidated, but one of the known causes is lipid accumulation in DCs in cancer patients, which may suppress DC function [21]. As we expect that elucidating the mechanism of lipid accumulation in DCs will lead to the development of new immunotherapy, we investigated the DC subsets and lipid accumulation in the peripheral blood of lung cancer patients to elucidate the changes in tumor immunity in lung cancer.

## 2. Methods

### 2.1. Patients

Subjects were treatment-naive lung cancer patients diagnosed histopathologically. Lung cancer disease staging was performed according to the 7th edition of the TNM Classification of Malignant Tumours [22]. The controls consisted of healthy individuals with no allergic disease, infection, or autoimmune disease and no history of malignant tumor. Informed consent was obtained from all subjects. The study was approved by the institutional ethics committee and was conducted in accordance with the ethical principles embodied in the Declaration of Helsinki.

### 2.2. Flow Cytometry

Twenty ml of heparinized peripheral blood was obtained from the controls and lung cancer patients. The following monoclonal antibodies were added to the fresh blood, which was cultured at room temperature for 2 h: Lineage-1, fluorescein isothiocyanate (FITC; BD Biosciences, San Jose, CA, USA); HLA-DR, Per-CP (BD Biosciences); CD11c, FITC (BD Biosciences); CD11c, PE (BD Biosciences); and CD123, PE (BD Biosciences). Thereafter, erythrocytes were hemolyzed by cell lysing solution (BD Biosciences). The DC subtype and percentage of each subtype were analyzed by flow cytometry (FACSCalibur, BD Biosciences). Data were analyzed using CellQuest Pro (BD Biosciences). After gating mononuclear cells based on side scatter and forward scatter, the blood DC population was identified as the lin^−^/HLA-DR^+^ fraction. DCs were divided into a CD11c^+^DC subset (mDCs) and a CD123^+^DC subset (pDCs). The number of total events was 200,000, and data are expressed as DC counts per 200,000 leukocytes.

### 2.3. Lipid Accumulation Analysis

Peripheral blood was collected from controls and lung cancer patients, and the Lineage(−) and HLA-DR(+) DC fractions were sorted using a FACSAria cell sorter (BD Biosciences) and stained with BODIPY 650/665 and DAPI (Polysciences, Warrington, PA, USA), a chromatin dye. The cells were analyzed by fluorescence microscopy for DC lipid accumulation. For the flow cytometry analysis, sorted DCs were immobilized and stained with BODIPY 650/665 at room temperature for 15 min.

### 2.4. Oil Red O Staining

Oil Red O (Sigma-Aldrich, St. Louis, MO, USA) is a type of azo dye, and because it is nonpolar and lipophilic, it is incorporated into intracellular lipids (i.e., triglycerides) and provides a means of specific triglyceride staining [23]. mDCs and pDCs were isolated from the peripheral blood of treatment-naive lung cancer patients and controls using the FACSAria, and slide specimens were made using a Cytospin. After fixation with 10% formalin, specimens were stained using Oil Red O, thus staining the intracellular triglycerides.

### 2.5. Quantification of DC Lipids

Intracellular triglycerides content in cell homogenate of DCs were measured using the Adipogenesis Assay Kit (BioVision, Milpitas, CA, USA), a high-sensitivity quantitation kit for intracellular triglycerides.

### 2.6. Mixed Lymphocyte Culture Reaction

T cell proliferation ability of DCs was evaluated by mixed leukocyte reaction. Peripheral blood was collected from lung cancer patients and controls. The Lineage(−), HLA-DR(+), CD11c(+), and CD123(−) mDC fractions were sorted using the FACSAria cell sorter and cocultured in 96-well plates with allogenic naive T cells at a ratio of 1 : 6. naive T cells were purified from PBMC by a negative selection method using naive CD4+ T cell isolation kit (Miltenyi Biotec, Bergisch Gladbach, Germany) according to the supplier's instructions. The purity of CD4^+^, CD45RA^+^, and CD45RO^−^ T cells was evaluated to be over 98% using flow cytometry. After 6 days of culture, BrdU (10 *μ*M 5-bromo-2′-deoxyuridine phosphate-buffered saline, pH 7.4; Roche Applied Science, Basel, Switzerland) was added, and the intracellular uptake of BrdU was conducted using a BrdU Cell Proliferation ELISA kit (Roche Applied Science) and measured using a microplate reader (Molecular Devices, Tokyo, Japan).

### 2.7. Statistics

Because the data obtained in this study were not normally distributed, we used nonparametric Wilcoxon test for comparisons between two groups and Kruskal–Wallis test for comparisons among multiple groups. Differences were considered significant for values of *p* < 0.05. Data are presented as the means ± SD. JMP Pro Cary (SAS Institute Inc., Cary, NC) was used for statistical analysis.

## 3. Results

### 3.1. Patient Background

Patient background is shown in Table 1. The mean age of the 29 lung cancer patients (21 men and 8 women) was 71.6 years (range: 53–84 years) and that of the 25 controls (10 men and 15 women) was 55.7 years (range: 28–67 years). Histological types of lung cancer were adenocarcinoma in 18 subjects, squamous cell carcinoma in 7 subjects, small-cell carcinoma in 3 subjects, and non-small-cell carcinoma in 1 subject. Clinical stage was I in 10 patients, II in 1 patient, III in 6 patients, and IV in 12 patients.

### 3.2. Number of Peripheral Blood DCs

**Figure 1:**
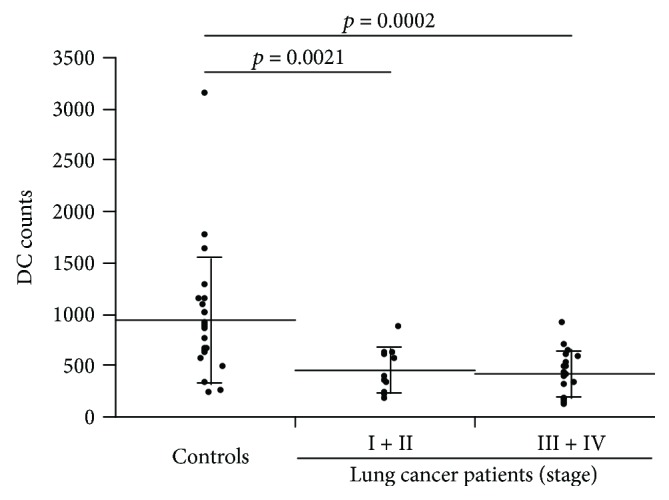
Peripheral blood dendritic cell (DC) count. After adding monoclonal antibody, peripheral blood samples were hemolyzed, and cell immobilization was conducted. HLA-DR(+) and Lineage-1(−) DC fractions were identified using a FACSCalibur, and the number of cells per 200,000 leukocytes was analyzed. Significant differences were observed between the stage I + II lung cancer patients and the controls (*p* = 0.0021) and between the stage III + IV lung cancer patients and the controls (*p* = 0.0002). Horizontal lines represent the means, and vertical lines represent the standard deviation.

**Figure 2:**
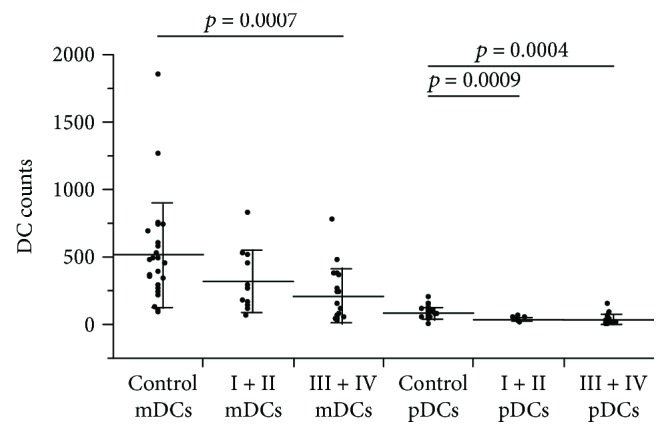
DC count by subtype. Lineage(−), HLA-DR(+), and CD11c(+) myeloid DC (mDC) and Lineage(−), HLA-DR(+), and CD123(−) plasmacytoid DC (pDC) fractions were counted and compared between the stage I + II and stage III + IV cancer patients. Significantly fewer mDCs were observed in stage III + IV cancer patients compared with the controls, and significantly fewer pDCs were observed in stage I + II and stage III + IV lung cancer patients compared with the controls.

Lung cancer patients had significantly fewer DCs (groups I + II: 452.6 ± 221.2/200,000 leukocytes, *p* = 0.0021; groups III + IV: 418.0 ± 219.9/200,000 leukocytes, *p* = 0.0002) than the controls (954.9 ± 629.2/200,000 leukocytes) (Figure 1). Patients with stage III + IV lung cancer had a significantly reduced number of mDCs (207.7 ± 201.3/200,000 leukocytes, *p* = 0.0007) than the controls (516.3 ± 386.8/200,000 leukocytes) (Figure 2). The number of pDCs in patients with stage I + II lung cancer (35.0 ± 14.9/200,000 leukocytes, *p* = 0.0009) and stage III + IV lung cancer (32.9 ± 38.8/200,000 leukocytes, *p* = 0.0004) was significantly lower than that in the controls (84.0 ± 45.8/200,000 leukocytes).

### 3.3. Lipid Accumulation in Peripheral Blood DC

**Figure 3:**
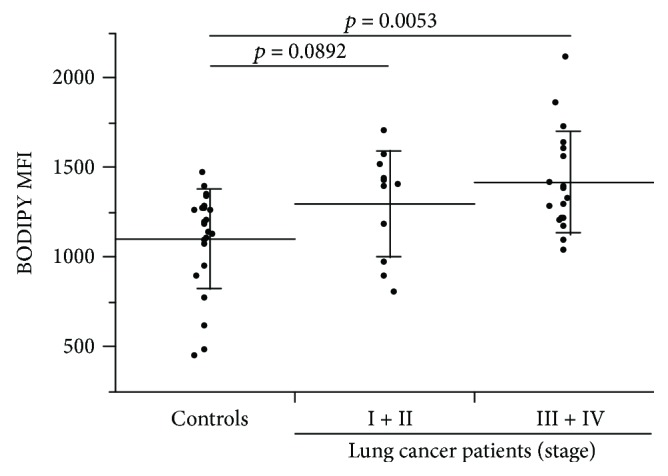
BODIPY 650/665 fluorescence intensity in peripheral blood DCs. The BODIPY 650/665 mean fluorescence intensity of HLA-DR(+) and Lineage-1(−) DC fractions was assessed in lung cancer patients according to clinical stages. A significant difference was observed between the stage III + IV lung cancer patients and the controls (*p* = 0.0053).

**Figure 4:**
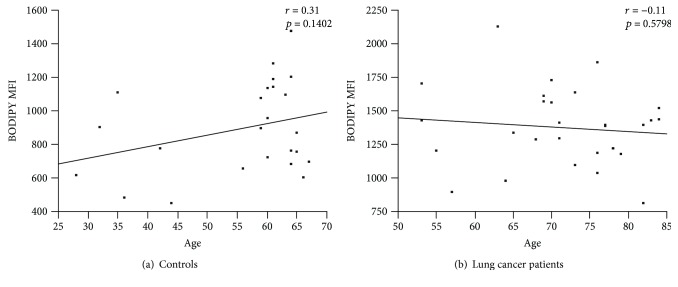
Correlation between age and lipid accumulation in DCs in the control group or lung cancer patient group. No significant correlation was observed between age and BODIPY MFI in DCs. The *r* value was 0.31 (*p* = 0.1402) in the control group (a) and −0.11 (*p* = 0.5798) in the lung cancer patient group (b).

**Figure 5:**
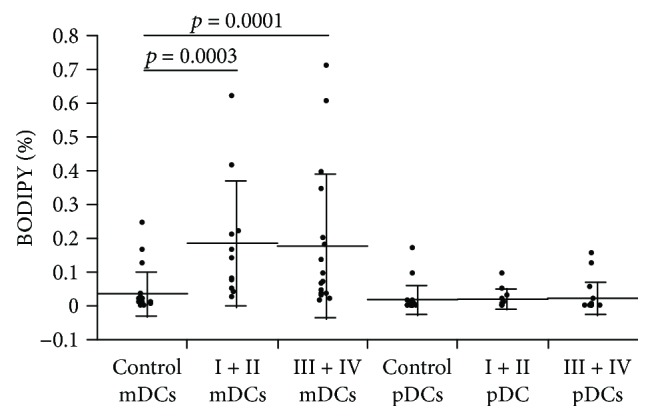
BODIPY 650/665 fluorescence intensity in mDC and pDC. This figure shows the percentages of HLA-DR(+), Lineage(−) DCs, and BODIPY-positive mDCs and pDCs. Significantly, more mDCs were observed in stage I + II and stage III + IV lung cancer patients than in the controls.

The mean fluorescence intensity (MFI) of peripheral blood DCs stained with BODIPY 650/665 was greater in patients with more advanced cancer stages than in the controls (1148.6 ± 237.1). The lipid accumulation in peripheral blood DCs was not significant in stage I + II patients (MFI, 1299.9 ± 293.8, *p* = 0.0892), but was significant in stage III + IV patients (MFI, 1419.7 ± 283.7, *p* = 0.0053) (Figure 3). There was no correlation between age and DC BODIPY 650/665 MFI in lung cancer patients or controls (Figure 4). The mDCs in stage I + II patients (0.185 ± 0.183%, *p* = 0.0003) and stage III + IV patients (0.177 ± 0.212%, *p* = 0.0001) were significantly elevated compared with the controls (0.036 ± 0.063%) (Figure 5). In contrast to mDCs, increased lipid accumulation was not seen in pDCs (Figure 5).

### 3.4. Fluorescence Microscopy for Intracellular Lipid Accumulation

**Figure 6:**
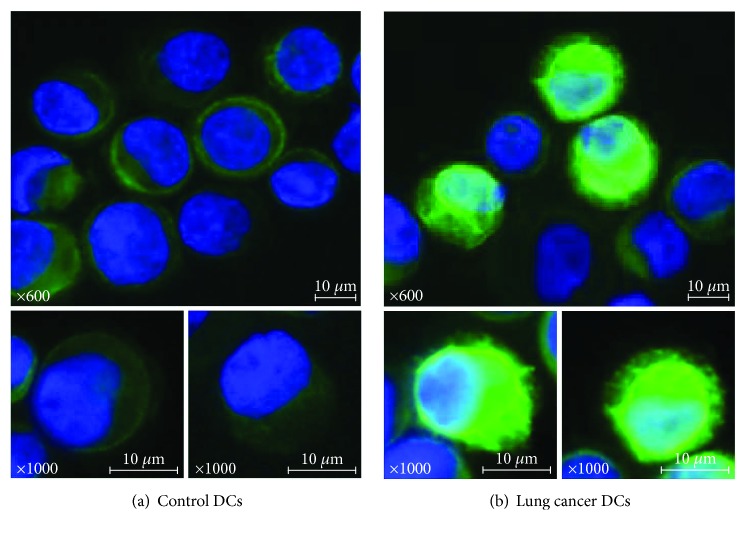
DC lipid accumulation. DCs isolated from the peripheral blood were stained using BODIPY 650/665 and confirmed under a light microscope. (a) Control DCs showing only slight fluorescence. (b) Stage IV lung cancer DCs showing strong fluorescence. Fluorescence microscopy photographs of DCs from representative cases were indicated.

Fluorescence microscopy photographs of DCs stained with BODIPY 650/665 from representative cases were shown in Figure 6. Control DCs showed only slight fluorescence. On the contrary, DCs from stage IV lung cancer patients showed strong and intense fluorescence indicating intracellular lipid accumulation.

### 3.5. Oil Red O Staining

**Figure 7:**
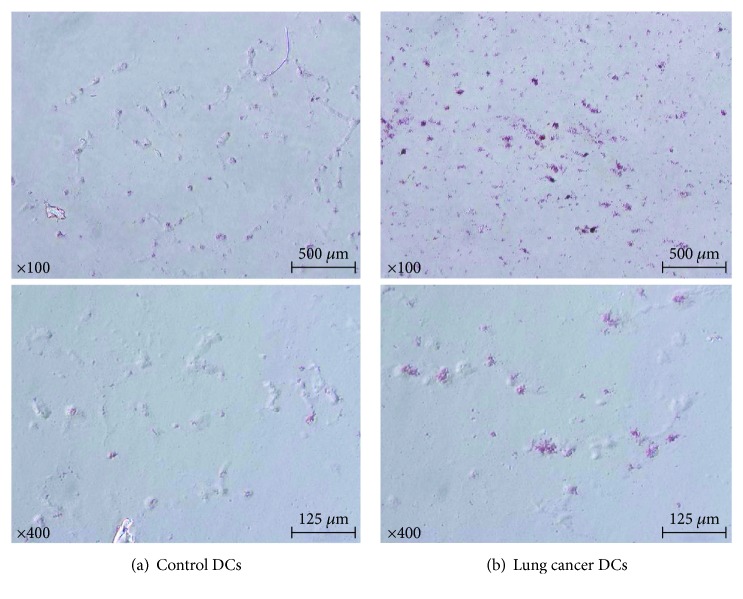
Oil Red O staining. Oil Red O staining is (a) low in the controls and (b) high in the stage IV lung cancer patients (representative cases).

More cells from stage IV lung cancer patients were stained with Oil Red O, suggesting that the intracellular lipids observed in BODIPY staining were triglycerides. DCs from representative cases were shown in Figure 7.

### 3.6. Quantitation of Intracellular Triglycerides in DCs

**Figure 8:**
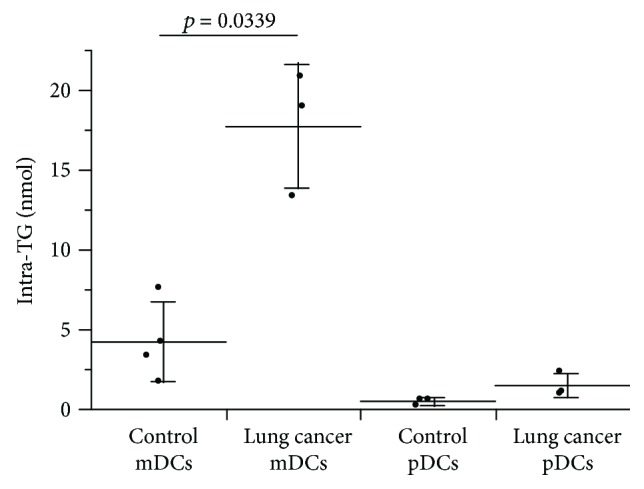
Quantitation of intracellular triglycerides in DCs. Lineage(−), HLA-DR(+), CD11c(+), and CD123(−) mDC fractions and Lineage(−), HLA-DR(+), and CD123(−) pDC fractions were sorted in the peripheral blood from the stage IV lung cancer patients and controls, and the cells were lysed to quantify the intracellular triglycerides. The intracellular triglyceride levels in mDCs were significantly higher in the lung cancer patients (*n* = 3) than in the controls (*n* = 4).

In some cases, intracellular triglycerides content were measured in homogenized DCs. The mDCs in stage IV lung cancer patients (*n* = 3) had significantly higher levels of triglycerides (17.73 ± 3.89 nmol, *p* = 0.0339) than those in the controls (*n* = 4, 4.23 ± 2.52 nmol). However, pDC triglyceride levels were low in both the lung cancer patients and the controls (Figure 8).

### 3.7. Mixed Lymphocyte Reaction (MLR)

**Figure 9:**
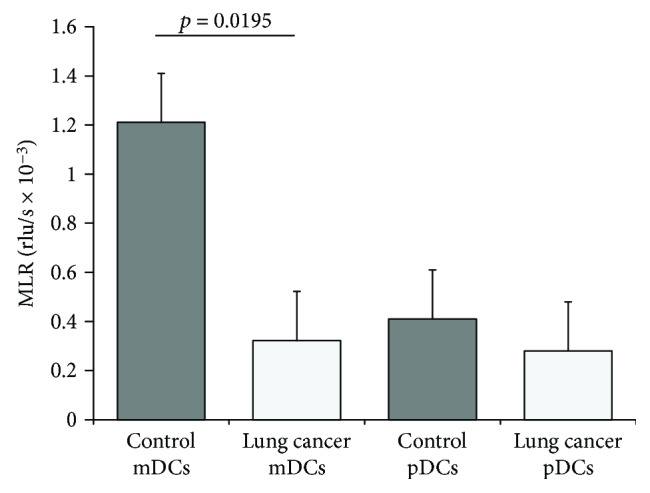
Mixed lymphocyte reaction (MLR). Peripheral blood DCs were cocultured with allogenic native T cells at a ratio of 1 : 6, and the rate of T cell proliferation was measured using BrdU. A lower MLR was observed in the stage IV lung cancer patient mDCs (*n* = 4) than in control mDCs (*n* = 4). These data represent means ± SEM of four independent experiments.

T cell proliferation ability of DCs which was evaluated by MLR was significantly lower in the mDCs from stage IV lung cancer patients (0.32 ± 0.26 rlu/s × 10^3^, *p* = 0.0195) than in those from the controls (1.21 ± 0.19 rlu/s × 10^3^) (Figure 9). In the case of pDCs, MLR was low in both the stage IV lung cancer patients and the controls.

## 4. Discussion

We confirmed increased lipid accumulation of triglycerides in the mDCs of lung cancer patients when assessing the DCs obtained from peripheral blood. Furthermore, this study showed for the first time an increase in DC lipid accumulation in line with cancer progression and metastasis. DCs with accumulated lipids are known to reduce lymphoproliferative ability, which in turn reduces anticancer immunity.

Lipids exist in various states, such as fatty acids, phospholipids, sphingolipids, sterols, and lipoproteins, and their metabolism and oxidation are known to influence immune cells. Recently, lipids have been reported to affect macrophage function, but few studies have reported the influence of lipids on DCs. Loscher et al. reported that conjugated linoleic acid suppresses the activity of nuclear factor-*κ*B (NF-*κ*B) and the production of IL-12, while inducing IL-10 via extracellular signal-regulated kinase in bone marrow-derived murine DCs [24]. In addition, Zapata-Gonzalez, et al. reported that fatty acids regulate the activity of human-derived DCs mainly via peroxisome proliferator-activated receptor-*γ* (PPAR-*γ*) [25]. Fatty acids are related to the maturity and function of DCs, and signal transmission may occur via membrane receptors (e.g., TLR), PPAR-*γ*, and NF-*κ*B.

In terms of the influence of lipid accumulation on DC function, the accumulation of intracellular lipids, particularly triglycerides, has been reported in murine DCs [21]. Furthermore, lipid accumulation has also been observed in DCs in non-small-cell lung cancer (NSCLC) and renal cell carcinoma. However, as these studies involved a small population of only 6 patients, further investigation was warranted. Therefore, we conducted the present study. By means of highly sensitive intracellular triglyceride quantitation and Oil Red O staining, we showed lipid accumulation of triglycerides in peripheral blood DCs in lung cancer patients, which supports the findings of Herber et al. [21]. In addition, our study made some new discoveries, such as that the number of DCs decreases and lipid accumulation by DCs is potentiated in line with cancer progression, and that lipid accumulation occurs in mDCs.

Because it was confirmed that accumulated lipids in the DCs obtained from lung cancer patients are mainly triglycerides from the results of Oil Red O staining and measurement of triglyceride content, dysfunction of DCs is thought to be attributed to the triglycerides. However, we cannot deny the participation of other lipids except triglycerides because we did not analyze all lipids.

Factors influencing lipid accumulation may include advanced age and serum triglyceride levels, as well as cancer stage. Although the mean age of the controls (55 years) was lower than that of the lung cancer patients, no correlation was observed between age and DC BODIPY 650/665 MFI in lung cancer patients, or controls, suggesting that age may not play a role. Moreover, no correlation was observed between serum triglyceride levels and DC BODIPY 650/665 MFI, suggesting that serum triglycerides may also not play a role (data not shown).

The receptors mediating DC lipid accumulation are scavenger receptors, and the expression of macrophage scavenger receptor 1 (Msr-1) is reported to increase in DCs with high lipid accumulation [21]. In our study, however, no increased expression of Msr-1 as a protein on the surface of peripheral blood DCs was observed in lung cancer patients (data not shown). Although expression of scavenger receptor B is potentiated during lipid accumulation in mouse bone marrow and spleen-derived DCs [26], the receptors mediating DC lipid accumulation may differ between mice and humans. Going forward, we need to elucidate the lipid accumulation mechanisms, including lipid synthesis, metabolism, and expressed receptors, in the DCs of lung cancer patients.

In the process of tumor immunity, DCs ingest cancer cell antigens and present them to T cells, which recognize the cancer cell antigens presented via the major histocompatibility complex (MHC) and induce apoptosis. Since we suspected a dysfunction of DCs in lung cancer patients during this process, we studied the mixed leukocyte reaction (MLR) of DCs in cancer patients. As the MLR response was reported to be high when the ratio of DCs to allogenic naive T cells was 1 : 6 [27], we used the same ratio in our study. The MLR using DCs with high levels of lipid accumulation in cancer patients was significantly lower than that in the controls, and DC lipid accumulation decreased antigen presentation to naive T cells, which was believed to suppress T cell-induced apoptosis. In addition, the decreased number of CD8(+) T cells observed in the peripheral blood of cancer patients (data not shown), and the possibly decreased induction and suppressed activation of CTL, may be influenced by factors such as TGF-*β* and IL-10 from cancer cells, as well as by DC lipid accumulation.

Various factors are reported to participate in decreased tumor immunity. For example, during long-term exposure to cancer antigens, expression of programmed death receptor-1 (PD-1), a costimulatory receptor, is observed in T cells, and the PD-1 ligand is expressed on cancer cells. Binding of these two components suppresses T cell activation [28, 29]. Development of anti-PD-1 antibodies results in competition for the PD-1 ligand binding site and may be a treatment strategy for overcoming T cell suppression and could therefore have clinical applications. The PD-1 antibodies have been already clinically used and placed as an important option of the lung cancer therapeutic drug [30–33]. The involvement of inhibitory and costimulatory markers other than PD-1, such as TIM-3, BTLA, and LAG-3, has also been reported [34–36].

Taken together, various mechanisms involved in tumor immunity cause immunosuppression and immunotolerance, thereby enabling cancer cells to evade tumor immunity. We focused on lipid accumulation in DCs and found a significant increase in lipid accumulation in mDCs associated with cancer progression. Reduced MLR levels were observed in DCs from cancer patients, thereby decreasing anticancer immune response. Going forward, it is necessary to elucidate the mechanism of lipid uptake by DCs to develop drugs that suppress lipid uptake. If this is achieved, a new form of immunotherapy to increase the anticancer immunity of cancer patients may become a reality.

## Figures and Tables

**Table 1:** Patient characteristics.

	Healthy volunteers	Lung cancer patients
Number	25	29
Age (range)	55.7 (28–67)	71.6 (53–84)
Gender (male/female)	10/15	21/8
Histology		
Adenocarcinoma		18
Squamous cell carcinoma		7
Small-cell carcinoma		3
NSCLC		1
Stage (I/II/III/IV)		10/1/6/12
